# Discovery Methodology of Novel Conotoxins from *Conus* Species

**DOI:** 10.3390/md16110417

**Published:** 2018-10-30

**Authors:** Ying Fu, Cheng Li, Shuai Dong, Yong Wu, Dongting Zhangsun, Sulan Luo

**Affiliations:** 1 Key Laboratory of Tropical Biological Resources, Ministry of Education, Hainan University, Haikou 570228, China; fuying926@163.com (Y.F.); licheng_0111@163.com (C.L.); dongshuai_1024@163.com (S.D.); wys211@163.com (Y.W.); zhangsundt@163.com (D.Z.); 2 Key Laboratory for Marine Drugs of Haikou, Hainan University, Haikou 570228, China

**Keywords:** discovery, methodology, novel conotoxins, crude venom purification, gene cloning, transcriptomics, proteomics, multi-omics

## Abstract

Cone snail venoms provide an ideal resource for neuropharmacological tools and drug candidates discovery, which have become a research hotspot in neuroscience and new drug development. More than 1,000,000 natural peptides are produced by cone snails, but less than 0.1% of the estimated conotoxins has been characterized to date. Hence, the discovery of novel conotoxins from the huge conotoxin resources with high-throughput and sensitive methods becomes a crucial key for the conotoxin-based drug development. In this review, we introduce the discovery methodology of new conotoxins from various *Conus* species. It focuses on obtaining full N- to C-terminal sequences, regardless of disulfide bond connectivity through crude venom purification, conotoxin precusor gene cloning, venom duct transcriptomics, venom proteomics and multi-omic methods. The protocols, advantages, disadvantages, and developments of different approaches during the last decade are summarized and the promising prospects are discussed as well.

## 1. Introduction

**Figure 1:**
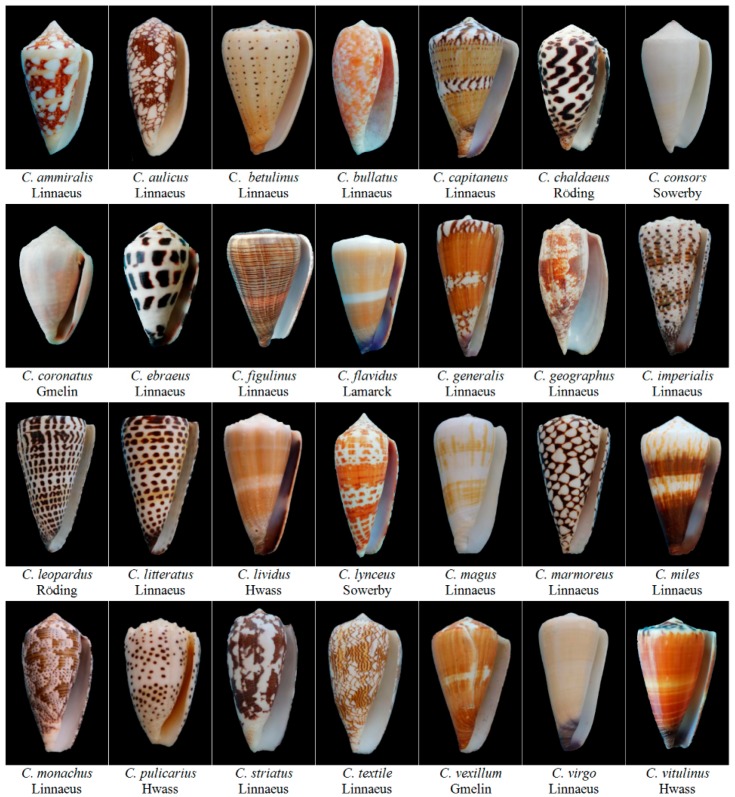
Representative *Conus* species native to Hainan China (shot by Cheng Li).

Cone snails (*Conus*) are carnivorous mollusks from the *Conidae* family (Figure 1). They live in the tropical oceans around the world and hunt fish (piscivorous), worms (vermivorous), or molluscs (molluscivorous) for food, although they are slow-moving creatures [1]. Cone snails have evolved a full set of specialized envenomation apparatus to release bioactive venoms to compensate their slow movement for fast-moving prey, competitors, or/and predators [2,3]. Cone snail venom peptides are secreted by the epithelial secretory cells in the long and convoluted venom duct [2,4]. The venom is pushed by muscle peristalsis of venom bulb and loaded into the harpoon-like radula tooth for envenomation [5]. Due to the human casualties that are caused by cone snail stings in 1960s [6], these venoms first caught researcher’s interest in their toxicity and bioactivity.

Early studies have confirmed that these bioactive venoms are a cocktail of neuroactive peptides, termed conopeptides or conotoxins, which can cause paralysis, shudder, and even death of the prey within seconds [1,5]. Subsequent research have revealed that conopeptides are able to selectively modulate voltage-gated ion channels [7] (Table 1), including sodium channels [8,9], potassium channels [10], and calcium channels [11,12], as well as ligand-gated ion channels (Table 1), such as nAChRs [13,14,15], serotonin receptor [16], NMDA receptor [17], GABA receptor [18], GPCRs [19] (α_1_-adrenoceptors [20,21], vasopressin receptor [22], neurotensin receptor [23]), and neurotransmitter transporters (noradrenaline transporter [21,24]), which are key targets for chronic diseases, like neuralgia [8,25,26], addiction [27], epilepsy [17,28], cancer [29], heart disease [30,31], and so on [32,33,34]. 

Additionally, the venom peptides show high selectivity and efficacy when interacting with the targets, resulting in minor side effects for disease treatment [35]. Hence, cone snail venoms provide an ideal resource for neuropharmacological tools and drug candidates screening, which have become a research hotspot in neuroscience and new drug development [36,37,38] (Table 1). For instance, an ω-conotoxin, named MVIIA (Ziconotide, Prialt) from *Conus magus*, which blocks voltage-gated calcium channels, has been approved by FDA for chronic pain treatment since 2004 [39,40]. At present, more than 10 conotoxins, including Xen2174 (MrIA) [41], CGX-1007 (Conantokin G) [17], CGX-1051 (κ-PVIIA) [42], ACV1 (Vc1.1) [43], and CGX-1160 (contulakin-G) [44] have marched into preclinical or clinical research stage, which present good prospects on conotoxin drug discovery.

There are more than 700 *Conu*s species in the world [48] and each can secrete over 1000 conotoxins [49]. In particular, 3305 novel conopeptide precursors were discovered from one *Conus episcopatus* specimen [50]. Owning to small overlap of conopeptides between different *Conus* species [51], there are an estimated 1,000,000 natural peptides that are produced by cone snails. However, <0.1% of the estimated conopeptides has been characterized to date [36,52]. Therefore, high-throughput and sensitive methods are crucial for the discovery of novel conotoxins and the development of conotoxin-based drug screening from this enormous peptide reservoir. 

In this review, the discovery methodology of novel conotoxins from mollusks *Conus* species has been summarized, which mainly focuses on obtaining full N- to C-terminal sequences, regardless of disulfide bond connectivity, through crude venom purification, conotoxin precusor gene cloning, venom duct transcriptomics, venom proteomics, and multi-omic method. The protocols, advantages, disadvantages, and developments of different approaches during the last ten years are overviewed and the promising prospects of these methods are discussed.

## 2. Diversity of Conotoxins

Conotoxins normally consist of 10 to 40 amino acid residues with 2 to 4 or more disulfide bonds. They are expressed as RNA-encoded precursor proteins, which are processed and transferred into mature peptides in the endoplasmic reticulum (ER) and Golgi apparatus. A typical conopeptide precursor is composed of a highly conserved ER signal region, a pro-region and a greatly variable mature peptide region [52]. Conotoxins can be classified into different gene superfamily categories, according to the similarities between the ER signal sequences [35].

Generally, conotoxin-encoding transcripts produce diverse precursors by hypermutation, fragment insertion/deletion, and mutation-induced premature termination [53]. One precursor can produce far more than one mature peptide because of various posttranslational modifications (PTMs) and variable peptide processing (VPP), which create the exponential diversity of conopetides [53,54]. For example, 20 different conopeptide variants on average for each precursor have been detected and characterized from venom duct transcriptomics of *Conus marmoreus* [49]. VPP refers to the C- and N-terminal truncations of the precursor by proteolytic cleavage at alternative sites [53,54]. These variations generated by interrupting, deleting, or elongating partial sequences and cysteine frameworks. It produced highly variable mature peptides or isoforms with multiple primary sequences [54]. 

PTMs are ubiquitous and play a key role in the structure and activity of conotoxins [55]. Many types of PTMs are found in the conotoxin maturation process, such as oxidative folding (disulfide bond formation, the most common PTMs), C-terminal amidation, hydroxylation of proline, valine, and lysine, carboxylation of glutamate, cyclization of N-terminal glutamine, glycosylation, sulfation, bromination, and residue epimerization, etc. [53,55]. These multi-diversification mechanisms, such as transcript variation, VPP and PTMs, explain how thousands of specific conopeptides are produced from such a limited gene precursors in a single *Conus* specie and reveal the reason for inter- and intra-specific variability [49,53,56].

## 3. Conotoxins Purified from Crude Venom

**Figure 2:**
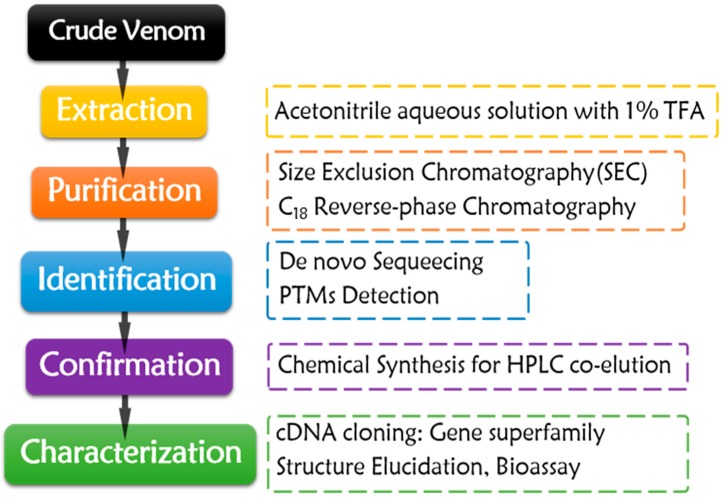
Purification workflow of native conotoxins obtained from crude venom.

Conotoxins have been obtained by isolation from crude venom of cone snails since 1970s [57] (Figure 2). The envenomation apparatus of the snails was dissected first. Only about 10 to 50 µL crude venom could be squeezed from each snail specimen, or the dissected tissues were directly subjected to extraction. Sometimes, tens to hundreds of collected snails were dissected to obtain enough venom for conopeptide isolation. The sampling process is non-renewable. It is a waste of precious resource of cone snails, especially for the rare species.

The purification process almost has remained constant for decades (Figure 2). Crude venom or dissected tissues are extracted by acetonitrile aqueous solution with 1% TFA. The crude extract is fractionated by Size Exclusion Chromatography (SEC), and then purified by C_18_ reverse-phase chromatography with gradient acetonitrile/water solution with 0.1% TFA as mobile phase. In order to gain enough target conopeptides for the subsequent characterization, enough crude venom and rigorous purification skills are required. The purified conotoxins are subjected to de novo sequencing through Edman degradation [58,59,60,61,62,63,64,65,66,67,68,69,70,71,72,73,74,75,76,77,78,79,80,81,82,83,84,85,86,87] or MS sequencing [81,88,89,90] after sequential disulfide bond reduction, hydrosulphonyl alkylation, and enzymolysis, which make sequencing process much easier. PTMs are assigned with the aid of MS techniques [59,65,66,69,70,71,75,76,77,78,80,83,89,90,91,92]. The targeted conotoxins are then chemically synthesized through SPPS, following the subsequent oxidative folding. HPLC co-elution of the synthesized peptides and the purified native conotoxins could validate the sequencing results [69,73,75,76,81,83,84,89]. For gene superfamily identification of the native peptides, their precursor genomic genes or cDNAs could be cloned by various PCR methods or identified by venom transcriptome sequencing. According to the signal peptide homology of the precursors, their gene superfamily could be determined and classified [31,62,65,68,74,75,79,82,86].

Conotoxins purified from *Conus* venom during the last ten years are summarized in Table 2. Fifty conotoxins in total were discovered during the last 10 years and only five in average were found and characterized per year, indicating that conotoxin discovery from crude venom isolation was stagnant. More efficient omic study is developing in full swing in recent years. It is difficult to isolate a novel conotoxin from limited amount of crude venom that consists of more than 1000 venom peptides. Blind search policy always makes the native peptide isolation process time-consuming and laborious. Therefore, only limited random conotoxins were discovered, which belonged to a few gene superfamilies (Table 2). Hence, more effective bioassay-guided and MS-sequence-tag guided fractionation methods are under development to facilitate the rapid discovery of novel native conotoxins from different crude venoms [62,86,87].

## 4. Gene Cloning to Discover New Conotoxins

To overcome the limitations of crude venom purification strategy, gene cloning for novel conotoxins discovery has emerged in 1990s [94]. Since a conotoxin is expressed by a specific gene, it can be amplified by PCR technique with specific primers [53,95,96,97,98]. 

**Figure 3:**
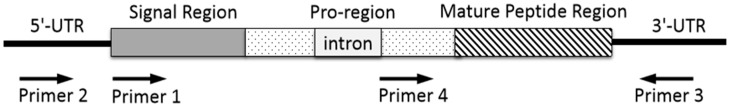
PCR amplification strategy to clone conotoxin precursor genes from genomic DNA.

Generally, genomic DNA is extracted from snail tissue of an individual specimen, or cDNA is prepared by reversed transcription of mRNA extracted from dissected venom duct. The resulting total DNA or cDNA is served as a template for PCR amplification with forward and reverse primers to perform 3′- and 5′-RACE. The primers are designed and synthesized on the basis of the conserved sequence in signal region (Figure 3, primer 1) or untranslated region of 3′- or 5′-UTRs (Figure 3, primer 2 and 3) of specific known conotoxin precursor, or its relatively conserved introns (Figure 3, primer 4). The PCR products are purified by electrophoresis on agarose gel and are ligated into a plasmid vector for sequencing. The annotation of possible conotoxin-encoding genes is conducted based on homologous searching. The resulting conotoxin sequences are analyzed and assigned by CLUSTALX [99]. The signal region sequences can be predicted by SignalP 3.0 server (http://www.cbs. dtu.dk/services/SignalP/) [99,100,101].

Primers make it possible for specific conotoxin-encoding genes to be amplified from the total genomic DNA or RNA of a cone snail. Thus the PCR primer design is a key factor for conotoxin discovery by gene cloning. Generally, the resulting PCR sequence of a conotoxin precursor gene generated by primer 1 or primer 2 pairing with primer 3, contains a complete open reading frame (ORF) sequence, which includes a signal region, a pro-region, and a mature peptide region (Figure 3). When primer 4 pairing with primer 3 is used to clone a conotoxin precursor gene, it starts with partial pro-region without signal peptide. Representative α-family (α*-) conotoxins discovered by gene cloning during the last ten years are shown in Table 3. The resulting sequences of Pu14.1 and GeXIVA consist of complete precursor sequences including signal regions which facilitate to assign gene superfamily category. Previous study showed that the sequences of the α-conotoxin intron in pro-region is highly conserved [97]. Many new α-conotoxins have been discovered by PCR technique using its conserved intron and 3′-UTR primers in our lab, such as α-conotoxin TxIB, TxID, LvIA, etc., which do not contain signal regions (Table 3). A forward primer and its paired reverse primer could be designed according to the conserved intron of a known gene superfamily and its 3′-UTR, such as A-, O-, or other superfamily, to clone novel conotoxin precursor genes. Random cDNA sequencing can also obtain the complete precursor sequence, e.g., VxXXIVA, but this method is not as targeted as the strategy with delicately designed primers. 

When compared with crude venom purification, the gene cloning strategy is more resource-saving. Generally, several or even one specimen is enough for conotoxin gene cloning. However, the mature peptide sequences are speculated from their precursor genes, so no PTMs identification is involved. On the other hand, gene cloning strategy is relatively low-throughput, when compared with the transcriptomic approach that arose in 2010s. In addition, the primers for gene cloning are designed according to the conserved sequences of known family or superfamily, so new family or superfamily conotoxins are difficult to be discovered by this way. 

## 5. Cone Snail Multi-Omics

Although big efforts have been made for novel conotoxin discovery from natural crude venom and gene cloning, most of the total estimated conotoxins have not been characterized yet [108]. More efficient, resource-saving, and high-throughput methodology urgently needs to be exploited. “Omics” such as transcriptomics and proteomics, and “Multi-omics” by integrating them together, have opened a new era for conotoxin discovery and rapidly accelerate the rate of conotoxin discovery [108,109,110].

### 5.1. Transcriptomics—A Useful Pathway to Identify Putative Conotoxins

Transcriptomics aims to identify and profile the holistic gene (including the conotoxin-encoding genes) transcription and expression at RNA level. Venom duct is an ideal material for transcriptomic analysis, because the number and level of conotoxin-encoding transcripts from venom duct are much larger than those from other tissues [50,111]. *Conus* venom duct transcriptomics is able to describe the conotoxin expression and it has presented a useful method to rapidly identify putative conopeptide sequences. In addition, transcriptomics using next generation sequencing (NGS) technology [112] makes large scale sequencing time- and cost-effective. 

**Figure 4:**
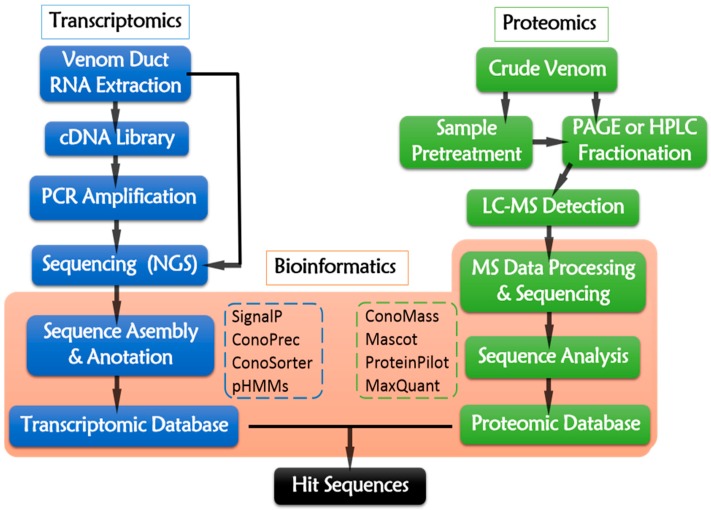
Multi-omic pipeline of conotoxin discovery.

The transcriptomic pipeline (Figure 4) starts from the total RNA extraction of dissected venom duct. Then, mRNAs are served as reverse-transcriptional templates for cDNA library construction. PCR amplification is conducted while using cDNA as template and specific sequences as primers. The resulting cDNA or the raw RNA sequences are sequencing using NGS platforms, such as 454 (Roche, Branford, CT, USA), Illumina (Illumina, San Diego, CA, USA), Ion Torrent Personal Genome Machine (Thermo Fisher, Waltham, MA, USA), Nanopore (Oxford, UK), ABI 3730 Series (Applied Biosystems, Foster City, CA, USA), and PacBio (Pacific Biosciences, Menlo Park, CA, USA) [108,113]. Illumina and Roche 454 are the most widely-used NGS platform at present (Table 4). The raw reads generated from NGS platforms require data assembly to remove artifacts, poor quality raw reads, as well as redundant and aberrant sequences [114]. The trimmed sequences are then deciphered into peptide primary sequences according to opening reading frames (ORFs) [112] by ConoPrec [1,53,54,111,115,116] or SignalP4.0 [115,116,117,118], which may locate the signal peptides and predict their cleavage sites. Profile Hidden Markov Models (pHMMs) [1,111,118,119] is a useful tool of ConoSorter [1,110,118,119], which could identify the putative precursors of conopeptides and categorize their superfamilies. Homology search and analysis by running BLAST against the combined searchable online databases, like ConoServer (The university of Queensland, Brisbane, Australia) (http://research1t.imb.uq.edu.au/conoserver/) [120,121], UniProtKB/Swiss-Prot (http://www.uniprot.org/downloads) [122,123], and NCBI (http://www.ncbi.nlm.nih.gov/), may enable the rapid identification of known and novel conotoxins. ConoSorter also facilitates to illustrating relative sequence frequency, length, number of cysteines, N-terminal hydrophobicity, and sequence similarity score [118]. Thus, a unique transcriptomic dataset for an individual specimen from a specific *Conus* specie might be established. 

Compared with traditional isolation and gene cloning, venom duct transcriptomic approach is a rapid, efficient, resource-saving, and high-throughput way to identify massive conotoxins from different cone snails, which greatly extends our cognition of conotoxin resource (Table 4). During the last decade, many putative conopeptide precursors have been identified from transcriptome of different *Conus* species (Table 4). At least 30 conopeptides precursors were discovered from *C. bullatus* by transcriptome sequencing. Surprisingly, as many as 3305 novel conopeptide precursors were discovered from a single *Conus episcopatus* specimen by sequencing its transcriptome (Table 4). 

Phylogeny-based conotoxin discovery utilizes the known conserved sequence to design specific primers for PCR amplification, which enables to find more conopeptides belonging to known superfamilies from different *Conus* species [124]. Additionally, specific PCR primers might be designed according to incomplete sequences that were obtained by MS-sequencing-tag or Edman degradation [124,125], which is also applied to clone new conotoxin precursors from venom transcriptome, cDNA, and its genomic DNA of various *Conus* species. It provides a feasible way to explore novel conotoxins belonging to new superfamilies [124]. cDNA library normalization is an effective and commonly-used method to equalize some specific cDNA, which facilitates to identify conotoxin genes with a relatively low level expression level [114,124]. Normalization suppresses highly abundant transcript reads and increases rare transcripts, so as to maximize the identified number of unique conotoxins [119].

Thanks to transcriptomic study, venom insulins, which target the heterospecific insulin receptors of prey, predators, and competitors, have been proven to be expressed in many worm- and snail-hunting cone snails [126]. Six insecticidal conotoxins have been validated and screened out from the transcriptomic dataset of 215 precursors by homologous search with α-conotoxin ImI [127]. These findings reveal that *Conus* transcriptomic database can promote the extension for new knowledge and find various new conopeptides.

### 5.2. Proteomics—An Effective Approach to Discovery Natural Conotoxins

Traditional proteomic identification depends on Edman degradation and amino acid composition analysis to assign the peptide sequences, but its sample-consuming and low-throughput characters make it difficult to be extensively applied. As the high-resolution MS instrument appears [135], venom proteomic study with the aid of modern MS technology has proven to be an effective and high-throughput approach for novel conotoxin discovery [108,109]. 

The general proteomic procedure is presented in Figure 4. Briefly, the venom sample is prepared by squeezing the venom from dissected venom duct (one-off operation), or collecting the secreted venom that is induced by pray from living cone snail individuals (reproducible operation) [1]. As the MS techniques develop, the required venom amount for experimental analysis of proteomics is decreased. Even about 7% or less of crude venom from one specimen is enough [136]. The proteomic data detected from different *Conus* species, especially for those cone snails hunting different preys, are quite different from each other, because different species and the food preference are the key factors for the evolution of venom diversity [137]. 

In traditional bottom-up proteomics, pretreatment of venom sample, such as reduction, alkylation, and enzymatic digestion, is carried out before HPLC-MS analysis in order to eliminate the influence of disulfide bonds, although it leads to partial loss of conopeptides during processing [50,53,111,115,138]. In top-down proteomic approach, intact disulfide-bridged venom peptides are remained, which makes it more applicable to analyze simple peptide mixtures, like highly purified venom subfractions, whereas bottom-up approach is more suitable for complex crude venom [50,53,139]. The combination of top-down and bottom-up approach enables the identification of several unexpected cleavage sites during conotoxin maturation [53]. 

The resulting vast MS data generated from LC-MS analysis are subjected to bioinformatic tools for further data processing and mining. Raw MS data are inputted into Mascot for Peptide mass fingerprint. ProteinPilot™ [1,49,54,115] is used for sequence identification and the annotation of precursor ions by searching the MS/MS mass list obtained at a relatively high precise level [54]. Parameters for enzymolysis and various types of PTMs are imported into ProteinPilot to identify PTMs and fragment splicing. ConoMass [49,115] and ProteinPilot are able to identify nearly all the PTMs except glycosylation, which requires assignment by de novo sequencing. The sequences are homologically searched and matched against databases, such as ConoServer, UniProtKB/Swiss-Prot, NCBI, and known transcriptomic dataset from its own, to identify the known and novel conopeptides as well as their gene superfamilies. The subsequent results are presented by various peptide sequences with a series of statistical data to profile the venom components.

Advanced mass analyzer, like TOF, especially Quadrupole-TOF (Q-TOF), shows rapid acquisition, high resolution, first-class sensitivity, and excellent mass accuracy. Ionization methods, such as ESI, MALDI, CID, ETD, EThcD, etc., provide options for obtaining alternative mass data for different purpose. ESI and MALDI are generally for proteomic study, whereas CID, ETD, EThcD are commonly for de novo MS sequencing by providing different dissociation patterns to acquire variable specific peptide fragments. More mature peptides can be detected by using superior MS instrument with advanced mass analyzer and efficient ionization technique. For instance, from venom proteomics of *Conus marmoreus*, there were 2710 peptide sequences revealed by MALDI-TOF; 3172 peptide sequences were detected by ESI-Q-TOF with regular electrospray; and 6254 peptide sequences were disclosed using ESI-Q-TOF, which is equipped with a DuoSpray ionization source [49]. ETD ionization strategy combined with targeted chemical derivatization has been applied to increase the charge state of conopeptides so as to maximize the detectable mass range, because the molecular masses of conotoxins usually exceed the optimum detective coverage [132]. Superior mass analyzer and various ionization methods are combined and applied to expand the boundary of accessible venom repertoire. Modern venom proteomics provides a methodology, not only for the rapid detection and characterization of specific conotoxins, but also for profiling an overview of the complex venom components. 

### 5.3. Bioinformaics—An Efficient Tool for Massive Data Processing and Integrating

Bioinformatics is an efficient tool for massive data processing and integrating, which has been deeply penetrative during the raw data processing, sequence identification, and superfamily classification by exquisite analytical softwares and algorithms with the introduction of integrated databases [108,140]. Venom duct transcriptomics and venom proteomics both benefit from the emergence and development of bioinformatics, especially the improvements on bioinformatic softwares, algorithms and expansion of searchable databases. The functions of the frequently-used tools for transcriptomics and proteomics are presented in Table 5. Transcriptomic and proteomic studies are quite reliant on the foundation database, which provides templates for sequence searching, matching, and annotating. The databases for sequence identification and BLAST should be the latest updated version, which should be composed of complete or partial natural precursor and mature toxin sequences generated either from conotoxin genes, transcripts, or proteins, as well as artificially synthesized conotoxins. Discovery of novel sequences using different approaches, in return, expands the capacity of the databases.

### 5.4. Multi-Omics Integration

A comprehensive strategy, named “multi-omics” or “venomics” [108,140], by integrating transcriptomics with proteomics through bioinformatics, is popular in the field of conotoxin research [110]. Although venom duct transcriptomics and venom proteomics both have proven to be effective and high-throughput methods to identify massive conopeptide sequences, the conotoxin sequences that are generated from transcriptomics are putative precursor peptides that need to be further confirmed for their real existence at protein level. Furthermore, no PTMs could be predicted from the precursor sequences. Luckily, venom proteomics is able to validate the putative peptides at protein level (Table 4) and identify nearly all the PTMs [49,128]. The validated sequencing and PTMs data can help to illustrate the processing mechanisms (transcript variation, VPP, PTMs) from precursor peptides (transcriptomic data) to the corresponding mature peptides (proteomic data).

Just as not every putative precursor can be validated by proteomic data, not all peptide sequences from proteomic data can find their corresponding precursors (Table 4). In fact, they were overlapped and matched with a very small percentage of 9.98% for *Conus episcopatus* [50]. The significant variations between the datasets of transcriptomics and proteomics actually exist, and the overlapped data (hit sequences) are not big enough. How to extend the datasets for making access to the completed repertoire of conotoxins? How to decode the variation so as to expand the overlapped or matched precursors with their corresponding mature peptides? Since one precursor can generate various mature conopeptides by the different PTMs. Theoretically, more mature peptides should be detected by proteomics. In practical use, the detected number always varies greatly with different MS instrument, bioinformatic analytical methods, fractionation, and sample pretreatment processes, etc. Rare transcripts at a low translational level are difficult to be recognized, which also contribute to the disparity. Thus, standardized processing protocols, reliable detection methods, dedicated integrated databases, and robust data analysis tools are needed. 

## 6. Conclusions and Prospects

In this review, we introduced the discovery methodology of novel conotoxins from various *Conus* species. It focused on obtaining full N- to C-terminal sequences, regardless of disulfide connectivity through crude venom purification, conotoxin precursor gene cloning, venom duct transcriptomics, venom proteomics, and multi-omic methods. The protocols, advantages, disadvantages, and developments of different approaches during the last decade and the promising prospects are summarized and discussed. To overcome the limitations of crude venom purification strategy, gene cloning technique have been developed and it temporarily slows down the deprivation of the native cone snail resource. 

In order to improve efficiency, high-throughput omic and multi-omic strategies have opened a new era for conotoxin discovery. Transcriptomics and proteomics are now acknowledged to be effective, resource-saving, and high-throughput approaches for novel conotoxin discovery. Multi-omic strategy is more efficient than using transcriptomics or proteomics alone. Efforts should be made to decode and reduce the variations between transcriptomic and proteomic data in order to expand the accessible repertoire of known conotoxins. The precursor processing mechanisms need to be illustrated as well. Thus, standardized processing protocols, reliable detection methods, dedicated integrated databases, and robust data analysis tools for transcriptomic, proteomic, and multi-omic studies are required to speed up novel conotoxin discovery.

## Figures and Tables

**Table 1:** Target and clinical potential of representative conotoxins.

Target/Mode of Action		Conotoxin	Clinical Potential	Ref.
Voltage-gated Ion Channels	Ca_v_ 2.2 inhibitor	MVIIA	Analgesia (On Market)	[40]
	Na_v_ 1.8 inhibitor	MrVIB	Analgesia	[45]
	K_v_ inhibitor	PVIIA	Cardiac reperfusion	[42]
Ligand-gated Ion Channels	α9α10 nAChRs inhibitor	Vc1.1	Analgesia (Phase II)	[43]
	NMDA-R inhibitor	Conantokin G	Analgesia/anti-epileptic	[17]
	5-HT_3_ inhibitor	GVIIIA	—	[16]
GPCRs	α_1_-adrenoceptor inhibitor	TIA	Cardiovascular/Benign Prostate Hyperplasia	[20,46]
	vasopressin receptor agonist	Conopressin-G	Cardiovascular/mood	[22]
	neurotensin receptor agonist	Contulakin-G	Analgesia (Phase Ia)	[23]
Neurotransmitter Transporters	noradrenaline transporter	MrIA	Analgesia (Phase I)	[47]

**Table 2:** Conotoxins isolated from cone snail venom during recent ten years.

Name	Species	Super-Family	Cystine Pattern	Sequence	Target/IC_50_	Year	Ref.
RegIIA	*C. regius*	A	I	GCCSHPACNVNNPHIC #	nAChR: α7/103 nM, α3β2/33 nM, α3β4/97 nM	2011	[60]
α-LsIA	*C. limpusi*	-	I	SGCCSNPACRVNNPNIC	nAChRs: α3β2/10 nM, α3α5β2/31 nM, α7/10 nM	2013	[87]
α-RgIB	*C. regius*	-	I	TWEECCKNPGCRNNHVDRCRGQV	α3β4 and/or α3β4α5 nAChRs	2013	[61]
α-BruIB	*C. brunneus*	-	I	DYCCRROTCIPIC #	Dα7 nAChR	2014	[62]
α-AusIA	*C. australis*	-	I	SCCARNPACRHNHPCV	α7 nAChR: 11.68 mM for AusIA (g), 9.67 mM for AusIA (r)	2014	[63]
Lo1a	*C. longurionis*	A	I	EGCCSNPACRTNHPEVCD	α7 nAChR/3.24 μM	2014	[64]
BnIA	*C. bandanus*	A	I	GCCSHPACSVNNPDIC #	α7 nAChR	2014	[65]
Im10A	*C. imperialis*	T	I	NTICCEGCMCY #	unknown	2016	[91]
α-EII_B_	*C. ermineus*	-	I	ZTOGCCWHPACGKNRC #	nAChRs	2017	[66]
PIC	*C. purpurascens*	A	I	SGCCKHPACGKNRC	rα1β1δε nAChR	2017	[67]
PIC[O7]				SGCCKHOACGKNRC			
lt3a	*C. litteratus*	M	III	DγCCγOQWCDGACDCCS	unknown	2009	[68]
κ-RIIIJ	*C. radiates*	M	III	LOSCCSLNLRLCOVOACKRNOCCT #	hK_v_1.2 channels/33 nM	2010	[69]
pr3a	*C. parius*	M	III	CCNWPCSFGCIPCCY	unknown	2010	[70]
pr3b				ERVCCGYOMSCKSRACKOSYCC #			
CnIIIC	*C. consors*	M	III	ZGCCNGPKGCSSKWCRDHARCC #	Na_v_1.4/1.3 nM α3β2 nAChR/450 nM	2012	[71]
BnIIID	*C. bandanus*	M	III	CCDBγNCDHLCSCCD #	unknown	2014	[72]
Asi3a	*C. asiaticus*	M	III	CCQWPCSHGCIPCCY #	unknown	2016	[91]
bt5a	*C. betulinus*	T	V	SγCCIRNFLCC	unknown	2010	[73]
pr6a	*C. parius*	O	VI/VII	TCLARDELCGASFLSNFLCCDGLCLLICV	unknown	2010	[70]
pr6b				FGSFIOCAHKGEOCTICCROLRCHEEKTOTCV			
pr6c				DQCTYCGIYCCPPKFCTSSGCRSP			
pr6d				YGNFOTCSETGEDCSAMHCCRSMTCRNNICAD			
MfVIA	*C. imperialis*	O	VI/VII	RDCQEKWEYCIVPILGFVYCCPGLICGPFVCV	Na_v_1.8/95.9 nM, Na_v_1.4/81 nM	2012	[88]
ge6b	*C. geneis*	O2O2	VI/VII	ACGGGGAPCGSSLDCCYPFECSYNSCG	unknown	2015	[74]
ge6c			VI/VII	ACGGGGAPCGSSLDCCYPFγCSYNSCG			
PiVIIA	*C. princeps*	O2	VI/VII	CDAOTHYCTNYWγCCSGYCγHSHCW	unknown	2016	[75]
vi6a	*C. virgo*	O1	VI/VII	DCGGQGEGCYTQOCCOGLRCRGGGTGGGVCQL	unknown	2016	[76]
Lo6/7a	*C. longurionis*	-	VI/VII	DQCSYCGIYCCPPKFCTSAGCRSP #	unknown	2016	[91]
Lo6/7b				SCLSSGALCGIDSNCCNGCNVPRNQCY #			
fu6a	*C. fulgetrum*	O	VI/VII	TCREKGEOCSVYVγCCSRICGYYACA	unknown	2016	[77]
α-GVIIIB	*C. geographus*	S	VIII	SGSTCTCFTSTNCQGSCECLSPPGCYCSNNGIRQPGCSCTCPGT #G	α9α10 nAChR/9.8 nM	2015	[92]
lt9a	*C. litteratus*	P	IX	IWFCASRTCSAPADCNPCTCESGVCVDWL	tetrodotoxin-sensi-tive sodium channels/300 nM	2017	[78]
lt9b				IWFCASRTCSAOADCNOCTCγSGVCVDWL	tetrodotoxin-sensi-tive sodium channels/504 nM		
Ca11a	*C. caracteristicus*	I	XI	AWPCGGVRASCSRHDDCCGSLCCFGTSTGCRVAVRPCW	unknown	2009	[79]
Ca11b				ALLCGGTHARCNRDNDCCGSLCCFGTCISAFVPC			
ts14a	*C. tessulatus*	A	XIV	DGCPPHPVPGMHPCMCTNTC	unknown	2010	[80]
Asi14a	*C. asiaticus*	-	XIV	SCGYPCSHCGIPGCYPG #	unknown	2016	[92]
pc16a	*C. pictus*	M	XVI	SCSCKRNFLCC #	unknown	2011	[81]
qc16a	*C. quercinus*	-	XVI	DCQPCGHNVCC	unknown	2011	[82]
αD-Ms	*C. mustelinus*	D	XX	DVRECQVNTPGSKWGKCCMTRMCGTMCCARSGCTCVYHWRRGHGCSCPG	nAChR: α7/0.12 nM, α3β2/1.08 nM, α4β2/4.5 nM	2009	[31]
αD-Cp	*C. capitaneus*	D	XX	EVQECQVDTPGSSWGKCCMTRMCGTMCCSRSVCTCVYHWRRGHGCSCPG	showed the same selectivity profile as αD-Ms, but has a lower potency		
α-GeXXA	*C. generalis*	D	XX	DVHRPCQSVRPGRVWGKCCLTRLCSTMCCARADCTCVYHTWRGHGCSCVM (dimer)	α9α10 nAChR	2015	[83]
im23a	*C. imperialis*	K	XXIII	IPYCGQTGAECYSWCIKQDLSKDWCCDFVKDIRMNPPADKCP	unknown	2012	[84]
im23b				IPYCGQTGAECYSWCIKQDLSKDWCCDFVKTIARLPPAHICSQ			
as25a	*C. cancellatus*	-	XXV	CKCPSCNFNDVTENCKCCIFRQP #	unknown	2013	[85]
as25b				CKCOSCNFNDVTENCKCCIFRQO?			
RsXXIVA	*C. regularis*	-	XXVI	CKGQSCSSCSTKEFCLSKGSRLMYDCCTGSCCGVKTAGVT	Ca_v_2.2	2013	[89]
GeXXVIIA	*C. generalis*	O	-	ALMSTGTNYRLLKTCRGSGRYCRSPYDCRRRYCRRISDACV	α9α10 nAChR/16.2 nM	2017	[93]
p21a	*C. purpurascens*	-	-	FELLPSQDRSCCIQKTLECLENYOGQASQRAHYCQQDATTNCODTYYFGCCPGYATCMSINAGNNVRSAFDKCINRLCFDPGH #	unknown	2011	[86]

#, [O], [γ], [B] represent C-terminal amidation, hydroxyproline, carboxyglutamate and bromotryptophan, respectively. Dα7 nAChR means the receptor is expressed in the CNS of the *Drosophila melanogaster* fly. The sequence of α-GeXXA (a dimer) presents one subchain of the dimer. ? indicates that the amidation of the C-terminus was not directly confirmed. Dash (-) means undetermined or none.

**Table 3:** Representative α*-conotoxins discovered by gene cloning during the last ten years.

Conotoxin	Super-Family	Primer	Sequence	Target (nAChRs)/IC_50_	Ref.
Pu14.1	A	signal sequence & 3′-UTR	MGMRMMFAVFLLVVLATTVVS*FNSDRASDGRNAAANVKASDLMARVLEK*DCPPHPVPGMHKCVCLKTC	rα1β1δε > rα6α3β2 > rα3β2	[73]
GeXIVA	O1	signal sequence	MKLTCVLIITVLFLTACQLTTA*VTYSRGEHKHRALMSTGTNYRLPK*TCRSSGRYCRSPYDRRRRYCRRITDACV	rα9α10/4.6 nM	[102]
TxIB	-	intron & 3′-UTR	*FDGRNTSANNKATDLMALPVR*GCCSDPPCRNKHPDLC #	rα6/α3β2β4/28 nM	[103]
TxID	-	intron & 3′-UTR	*FDGRNAAGNDKMSALMALTTR*GCCSHPVCSAMSPIC	rα3β4/12.5 nM, rα6/α3β4/94 nM	[104]
LvIA	-	intron & 3′-UTR	*FRGRDAAAKASGLVGLTDRR*GCCSHPACNVDHPEIC #	rα3β2 (8.7 nM) > rα6/α3β2β3 ≈ rα6/α3β4 ≈ rα3β4 > α7	[105]
Lt1.3	-	intron & 3′-UTR	*FDGRNAAPSDKASDLISLAVR*GCCSHPACSGNNPYFC #	α3β2/44.8 nM	[106]
VxXXIVA	B	cDNA sequencing	METLTLLWRASSSCLLVVLSHSLLRLLGVRCLEKSGAQPNKLFRPPCCQKGPSFARHSRCVYYTQSRE	rα9α10/1.2 μM, Mouse α1β1γδ/6.6 μM	[107]

The signal region is shadowed. The pro-region is italics. The mature conotoxin sequence is underlined. # represents C-terminal amidation. “r” indicates rat.

**Table 4:** The reported transcriptomic and proteomic data from various cone snails during the past decade.

Species	Number of Precursors	Number of Gene Superfamily	Sequencing Platforms	Number of Confirmed Conotoxins by Proteomics	MS Instruments	Year	Ref.
*C. textile*	-	-	-	31	ESI-LTQ-Orbitrap	2010	[128]
*C. bullatus*	30	6	Illumina, Roche 454	-	-	2011	[129]
*C. consors*	53	11	Roche 454	-	-	2012	[130]
*C. pulicarius*	82 (79 new)	14	Roche 454	-	-	2012	[131]
*C. marmoreus*	105	13	Roche 454	2710–6254	MALDI-TOF, ESI-Q-TOF	2013	[49]
*C. marmoreus*	158	13 new	Roche454	106	ESI-MS/MS	2013	[118]
*C. miles*	662	16 (8 new)	Roche 454	48	ESI-Q-TOF	2013	[54]
*C. flavidus*	-	-	-	31	ESI-LTQ-Orbitrap	2013	[53]
*C. victoriae*	113	20	Roche454	-	-	2014	[119]
*C. geographus*	127	16 (4 new)	Roche454	43	ESI-TripleTOF	2014	[3]
*C. catus*	104	11	Roche 454	51	ESI-Q-TOF	2015	[115]
*C. episcopatus*	3305	25 (16 new)	Illumina	1,448	ESI-MS/MS ESI-Q-TOF	2015	[50]
*C. tribblei*	136	30 (6 new)	Illumina, Roche 454	-	-	2015	[132]
*C. tribblei* *C. lenavati*	100 (45 new) 132	39 40	ABI 3730XL	-	-	2015	[116]
*C. planorbis*	182	25	Roche 454	23	ESI-TripleTOF	2015	[133]
*C. betulinus*	215 (183 new)	9 new	Illumina	-	-	2016	[111]
*C. vexillum* *C. capitaneus*	220	19 (4 new)	Roche 454	24	ESI-Q-TOF, MALDI-TOF	2016	[1]
*C. gloriamaris*	108 (98 new)	31	Illumina	-	-	2017	[134]

Dash (-) means undetermined.

**Table 5:** Frequently-used bioinformatic tools for cone snail venom transcriptomics and proteomics.

Tool	Developer	Function
*Tools for transcriptomics*		
SignalP	Technical University of Denmark, Denmark	Predict and locate the signal peptides and their cleavage sites
ConoPrec	The university of Queensland, Australia	Identify ORF and analyze contigs coding for conopeptide precursors, predict signal peptides and their cleavage site; Superfamily categorization
ConoSorter	—	Identify and classify precursor conotoxins into gene superfamilies; Provide relevant information (frequency of protein sequences, length, number of cysteine residues, hydrophobicity rate of N-terminal region etc.)
pHMMs	Technical University of Denmark, Denmark	Identify precursor peptides and classify the sequences into gene superfamily
*Tools for proteomics*		
ConoMass	The university of Queensland, Australia	Match experimental proteomic mass list against the mass predicted from transcripts, mass spectrometry comparison; PTMs identification
Mascot	Mascot science, UK	Peptide mass fingerprint; MS/MS database searches
ProteinPilot	AB SCIEX, USA	Searching and identification of mass sequences; Identification of PTMs
MaxQuant	Max Planck Institute of Biochemistry, Germany	Quantitative analysis of label-free and SILAC-based analysis; PTMs identification

## Abbreviations

BLAST	Basic local alignment search tool
CID	Collision induced dissociation
ESI	Electrospray ionization
ETD	Electron transfer dissociation
EThcD	Electron transfer higher energy collision dissociation
GPCRs	G protein-coupled receptors
HPLC	High performance liquid chromatography
LTQ-Orbitrap	Linear Trap Quatropole-Orbitrap
MS	Mass spectrometry
MALDI	Matrix-assisted laser desorption ionization
nAChRs	Nicotinic acetylcholine receptors
NCBI	National center of biotechnology information
NMR	Nuclear magnetic resonance
NMDA	*N*-methyl-*d*-aspartic acid receptor
PAGE	Poly acrylamide gel electrophoresis
PCR	Polymerase chain reaction
RACE	Rapid amplification of cDNA ends
SILAC	Stable isotope labeling by amino acids in cell culture
SPPS	Solid phase peptide synthesis
TOF	Time of flight

## References

[1] Prashanth J.R., Dutertre S., Jin A.H., Lavergne V., Hamilton B., Cardoso F.C., Griffin J., Venter D.J., Alewood P.F., Lewis R.J. (2016). The role of defensive ecological interactions in the evolution of conotoxins. Mol. Ecol..

[2] Endean R., Duchemin C. (1967). The venom apparatus of *Conus magus*. Toxicon.

[3] Dutertre S., Jin A.H., Vetter I., Hamilton B., Sunagar K., Lavergne V., Dutertre V., Fry B.G., Antunes A., Venter D.J. (2014). Evolution of separate predation- and defence-evoked venoms in carnivorous cone snails. Nat. Commun..

[4] Marshall J., Kelley W.P., Rubakhin S.S., Bingham J.P., Sweedler J.V., Gilly W.F. (2002). Anatomical correlates of venom production in *Conus californicus*. Biol. Bull..

[5] Salisbury S.M., Martin G.G., Kier W.M., Schulz J.R. (2010). Venom kinematics during prey capture in *Conus*: The biomechanics of a rapid injection system. J. Exp. Biol..

[6] Kohn A.J. (1958). Cone Shell Stings. Recent Cases of Human Injury due to Venomous Marine Snails of the Genus *Conus*. Hawaii Med. J..

[7] Terlau H., Olivera B.M. (2004). *Conus* venoms: A rich source of novel ion channel-targeted peptides. Physiol. Rev..

[8] Tosti E., Boni R., Gallo A. (2017). µ-Conotoxins Modulating Sodium Currents in Pain Perception and Transmission: A Therapeutic Potential. Mar. Drugs.

[9] Oliver K., Mcarthur J.R., Adams D.J. (2012). Conotoxins Targeting Neuronal Voltage-Gated Sodium Channel Subtypes: Potential Analgesics?. Toxins.

[10] Leipold E., Ullrich F., Thiele M., Tietze A.A., Terlau H., Imhof D., Heinemann S.H. (2017). Subtype-specific block of voltage-gated K^+^ channels by μ-conopeptides. Biochem. Biophys. Res. Commun..

[11] Ramírez D., Gonzalez W., Fissore R.A., Carvacho I. (2017). Conotoxins as Tools to Understand the Physiological Function of Voltage-Gated Calcium (Ca_V_) Channels. Mar. Drugs.

[12] Bourinet E., Zamponi G.W. (2017). Block of voltage-gated calcium channels by peptide toxins. Neuropharmacology.

[13] Giribaldi J., Dutertre S. (2018). α-Conotoxins to explore the molecular, physiological and pathophysiological functions of neuronal nicotinic acetylcholine receptors. Neurosci. Lett..

[14] Dutertre S., Nicke A., Tsetlin V.I. (2017). Nicotinic acetylcholine receptor inhibitors derived from snake and snail venoms. Neuropharmacology.

[15] Azam L., Mcintosh J.M. (2009). Alpha-conotoxins as pharmacological probes of nicotinic acetylcholine receptors. Acta Pharmacol. Sin..

[16] England L.J., Imperial J., Jacobsen R., Craig A.G., Gulyas J., Akhtar M., Rivier J., Julius D., Olivera B.M. (1998). Inactivation of a serotonin-gated ion channel by a polypeptide toxin from marine snails. Science.

[17] Barton M.E., White H.S., Wilcox K.S. (2004). The effect of CGX-1007 and CI-1041, novel NMDA receptor antagonists, on NMDA receptor-mediated EPSCs. Epilepsy Res..

[18] Castro J., Harrington A.M., Garciacaraballo S., Maddern J., Grundy L., Zhang J., Page G., Miller P.E., Craik D.J., Adams D.J. (2017). α-Conotoxin Vc1.1 inhibits human dorsal root ganglion neuroexcitability and mouse colonic nociception via GABAB receptors. Gut.

[19] Daniel J.T., Clark R.J. (2017). G-Protein Coupled Receptors Targeted by Analgesic Venom Peptides. Toxins.

[20] Chen Z., Rogge G., Hague C., Alewood D., Colless B., Lewis R.J., Minneman K.P. (2004). Subtype-selective noncompetitive or competitive inhibition of human alpha1-adrenergic receptors by rho-TIA. J. Biol. Chem..

[21] Sharpe I.A., Gehrmann J., Loughnan M.L., Thomas L., Adams D.A., Atkins A., Palant E., Craik D.J., Adams D.J., Alewood P.F. (2001). Two new classes of conopeptides inhibit the alpha1-adrenoceptor and noradrenaline transporter. Nat. Neurosci..

[22] Möller C., Marí F. (2007). A vasopressin/oxytocin-related conopeptide with gamma-carboxyglutamate at position 8. Biochem. J..

[23] Lee H.K., Zhang L., Smith M.D., Walewska A., Vellore N.A., Baron R., Mcintosh J.M., White H.S., Olivera B.M., Bulaj G. (2015). A marine analgesic peptide, Contulakin-G, and neurotensin are distinct agonists for neurotensin receptors: Uncovering structural determinants of desensitization properties. Front. Pharmacol..

[24] Paczkowski F.A., Sharpe I.A., Dutertre S., Lewis R.J. (2007). chi-Conotoxin and tricyclic antidepressant interactions at the norepinephrine transporter define a new transporter model. J. Biol. Chem..

[25] Romero H.K., Christensen S.B., Di Cesare Mannelli L., Gajewiak J., Ramachandra R., Elmslie K.S., Vetter D.E., Ghelardini C., Iadonato S.P., Mercado J.L. (2017). Inhibition of alpha9alpha10 nicotinic acetylcholine receptors prevents chemotherapy-induced neuropathic pain. Proc. Natl. Acad. Sci. USA.

[26] Hannon H.E., Atchison W.D. (2013). Omega-Conotoxins as Experimental Tools and Therapeutics in Pain Management. Mar. Drugs.

[27] Crooks P.A., Bardo M.T., Dwoskin L.P. (2014). Nicotinic receptor antagonists as treatments for nicotine abuse. Adv. Pharmacol..

[28] Gandini M.A., Sandoval A., Felix R. (2015). Toxins targeting voltage-activated Ca2^+^ channels and their potential biomedical applications. Curr. Top. Med. Chem..

[29] Irasema O.P., Mario N., Cervantes-Luevano K.E., Carolina Á.-D., Guy S., Sanchez-Campos L.N., Licea-Navarro A.F. (2016). Apoptosis Activation in Human Lung Cancer Cell Lines by a Novel Synthetic Peptide Derived from *Conus californicus* Venom. Toxins.

[30] Yang R., Liu Y., Hou X., Fan Y., Li J., Chen M., Wang Y., Zhang X., Zhang M. (2018). MAPKs-mediated modulation of the myocyte voltage-gated K^+^ channels is involved in ethanol-induced rat coronary arterial contraction. Eur. J. Pharmacol..

[31] Chen P., Dendorfer A., Finolurdaneta R.K., Terlau H., Olivera B.M. (2010). Biochemical Characterization of κM-RIIIJ, a Kv1.2 Channel Blocker. J. Biol. Chem..

[32] Vetter I., Lewis R.J. (2012). Therapeutic potential of cone snail venom peptides (conopeptides). Curr. Top. Med. Chem..

[33] Lewis R.J., Dutertre S., Vetter I., Christie M.J. (2012). *Conus* venom peptide pharmacology. Pharmacol. Rev..

[34] Layer R.T., Mcintosh J.M. (2006). Conotoxins: Therapeutic Potential and Application. Mar. Drugs.

[35] Akondi K.B., Muttenthaler M., Dutertre S., Kaas Q., Craik D.J., Lewis R.J., Alewood P.F. (2014). Discovery, synthesis, and structure: Activity relationships of conotoxins. Chem. Rev..

[36] Gao B., Peng C., Yang J., Yi Y., Zhang J., Shi Q. (2017). Cone Snails: A Big Store of Conotoxins for Novel Drug Discovery. Toxins.

[37] Prashanth J.R., Brust A., Jin A.H., Alewood P.F., Dutertre S., Lewis R.J. (2014). Cone snail venomics: From novel biology to novel therapeutics. Future Med. Chem..

[38] Halai R., Craik D.J. (2009). Conotoxins: Natural product drug leads. Nat. Prod. Rep..

[39] Miljanich G.P. (2004). Ziconotide: Neuronal calcium channel blocker for treating severe chronic pain. Curr. Med. Chem..

[40] Pope J.E., Deer T.R. (2013). Ziconotide: A clinical update and pharmacologic review. Expert Opin. Pharmacother..

[41] Obata H., Conklin D., Eisenach J.C. (2005). Spinal noradrenaline transporter inhibition by reboxetine and Xen2174 reduces tactile hypersensitivity after surgery in rats. Pain.

[42] Lubbers N.L., Campbell T.J., Polakowski J.S., Bulaj G., Layer R.T., Moore J., Gross G.J., Cox B.F. (2005). Postischemic administration of CGX-1051, a peptide from cone snail venom, reduces infarct size in both rat and dog models of myocardial ischemia and reperfusion. J. Cardiovasc. Pharm..

[43] Clark R.J., Fischer H., Nevin S.T., Adams D.J., Craik D.J. (2006). The synthesis, structural characterization, and receptor specificity of the alpha-conotoxin Vc1.1. J. Biol. Chem..

[44] Kern S., Allen J., Wagstaff J.S., Yaksh T. (2007). The pharmacokinetics of the conopeptide contulakin-G (CGX-1160) after intrathecal administration: An analysis of data from studies in beagles. Anesth. Analg..

[45] Wilson M.J., Zhang M.M., Azam L., Olivera B.M., Bulaj G., Yoshikami D. (2011). Navβ subunits modulate the inhibition of Nav1.8 by the analgesic gating modifier μO-conotoxin MrVIB. J. Pharmacol. Exp. Ther..

[46] Hieble J.P., Robert R.R. (1996). The use of alpha-adrenoceptor antagonists in the pharmacological management of benign prostatic hypertrophy: An overview. Pharmacol. Res..

[47] Brust A., Palant E., Croker D.E., Colless B., Drinkwater R., Patterson B., Schroeder C.I., Wilson D., Nielsen C.K., Smith M.T. (2009). chi-Conopeptide pharmacophore development: Toward a novel class of norepinephrine transporter inhibitor (Xen2174) for pain. J. Med. Chem..

[48] Puillandre1 N., Duda T.F., Meyer C., Olivera B.M., Bouchet P. (2015). One, four or 100 genera? A new classification of the cone snails. J. Molluscan Stud..

[49] Dutertre S., Jin A.H., Kaas Q., Jones A., Alewood P.F., Lewis R.J. (2013). Deep venomics reveals the mechanism for expanded peptide diversity in cone snail venom. Mol. Cell. Proteom..

[50] Lavergne V., Harliwong I., Jones A., Miller D., Taft R.J., Alewood P.F. (2015). Optimized deep-targeted proteotranscriptomic profiling reveals unexplored *Conus* toxin diversity and novel cysteine frameworks. Proc. Natl. Acad. Sci. USA.

[51] Davis J., Jones A., Lewis R.J. (2009). Remarkable inter- and intra-species complexity of conotoxins revealed by LC/MS. Peptides.

[52] Woodward S.R., Cruz L.J., Olivera B.M., Hillyard D.R. (1990). Constant and hypervariable regions in conotoxin propeptides. EMBO J..

[53] Lu A., Yang L., Xu S., Wang C. (2014). Various Conotoxin Diversifications Revealed by a Venomic Study of *Conus flavidus*. Mol. Cell. Proteom..

[54] Jin A.H., Dutertre S., Kaas Q., Lavergne V., Kubala P., Lewis R.J., Alewood P.F. (2013). Transcriptomic messiness in the venom duct of *Conus miles* contributes to conotoxin diversity. Mol. Cell. Proteom..

[55] Jakubowski J.A., Kelley W.P., Sweedler J.V. (2006). Screening for post-translational modifications in conotoxins using liquid chromatography/mass spectrometry: An important component of conotoxin discovery. Toxicon.

[56] Riveraortiz J.A., Cano H., Marí F. (2011). Intraspecies variability and conopeptide profiling of the injected venom of *Conus ermineus*. Peptides.

[57] Cruz L.J., Gray W.R., Olivera B.M. (1978). Purification and properties of a myotoxin from *Conus geographus* venom. Arch. Biochem. Biophys..

[58] Edman P., Begg G. (1967). A Protein Sequenator. FEBS J..

[59] Wang L., Liu J., Pi C., Zeng X., Zhou M., Jiang X., Chen S., Ren Z., Xu A. (2009). Identification of a novel M-superfamily conotoxin with the ability to enhance tetrodotoxin sensitive sodium currents. Arch. Toxicol..

[60] Van D.H.A., Peigneur S., Dyubankova N., Möller C., Marí F., Diego-García E., Naudé R., Lescrinier E., Herdewijn P., Tytgat J. (2012). Pc16a, the first characterized peptide from *Conus pictus* venom, shows a novel disulfide connectivity. Peptides.

[61] Bernáldez J., Romángonzález S.A., Martínez O., Jiménez S., Vivas O., Arenas I., Corzo G., Arreguín R., García D.E., Possani L.D. (2013). A *Conus regularis* Conotoxin with a Novel Eight-Cysteine Framework Inhibits Ca_V_2.2 Channels and Displays an Anti-Nociceptive Activity. Mar. Drugs.

[62] Lebbe E.K., Peigneur S., Maiti M., Mille B.G., Devi P., Ravichandran S., Lescrinier E., Waelkens E., D’Souza L., Herdewijn P. (2014). Discovery of a new subclass of α-conotoxins in the venom of *Conus australis*. Toxicon.

[63] Lebbe E.K.M., Peigneur S., Maiti M., Devi P., Ravichandran S., Lescrinier E., Ulens C., Waelkens E., D’Souza L., Herdewijn P. (2014). Structure-Function Elucidation of a New α-Conotoxin, Lo1a, from *Conus longurionis*. J. Biol. Chem..

[64] Nguyen B., Le C.J., Aráoz R., Thai R., Lamthanh H., Benoit E., Molgó J. (2014). Isolation, purification and functional characterization of alpha-BnIA from *Conus bandanus* venom. Toxicon.

[65] Xu S., Zhang T., Kompella S.N., Yan M., Lu A., Wang Y., Shao X., Chi C., Adams D.J., Ding J. (2015). Conotoxin alphaD-GeXXA utilizes a novel strategy to antagonize nicotinic acetylcholine receptors. Sci. Rep..

[66] Lei W., Liu J., Ren Z., Yu C., Xu A. (2017). Discovery of two P-superfamily conotoxins, lt9a and lt9b, with different modifications on voltage-sensitive sodium channels. Toxicon.

[67] Jiang S., Tae H.S., Xu S., Shao X., Adams D.J., Wang C. (2017). Identification of a Novel O-Conotoxin Reveals an Unusual and Potent Inhibitor of the Human α9α10 Nicotinic Acetylcholine Receptor. Mar. Drugs.

[68] Yuan D.D., Liu L., Shao X.X., Peng C., Chi C.W., Guo Z.Y. (2009). New conotoxins define the novel I3-superfamily. Peptides.

[69] Ni H., Chen F., Cai H., Xiao A., You Q., Lu Y. (2010). Isolation and Characterization of Conotoxin bt5a from *Conus betulinus*. Chin. J. Nat. Med..

[70] Möller C., Marí F. (2011). 9.3 KDa components of the injected venom of *Conus purpurascens* define a new five-disulfide conotoxin framework. Biopolymers.

[71] Aguilar M.B., Zugasti-Cruz A., Falcón A., Batista C.V.F., Olivera B.M., Cotera E.P.H.D.L. (2013). A novel arrangement of Cys residues in a paralytic peptide of *Conus cancellatus* (jr. syn.: *Conus austini*), a worm-hunting snail from the Gulf of Mexico. Peptides.

[72] Heghinian M.D., Mejia M., Adams D.J., Godenschwege T.A., Marí F. (2015). Inhibition of cholinergic pathways in Drosophila melanogaster by α-conotoxins. FASEB J..

[73] Peng C., Ye M., Wang Y., Shao X., Yuan D., Liu J., Hawrot E., Wang C., Chi C. (2010). A new subfamily of conotoxins belonging to the A-superfamily. Peptides.

[74] Christensen S.B., Bandyopadhyay P.K., Olivera B.M., McIntosh J.M. (2015). αS-conotoxin GVIIIB potently and selectively blocks α9α10 nicotinic acetylcholine receptors. Biochem. Pharmacol..

[75] Espino S.S., Dilanyan T., Imperial J.S., Aguilar M.B., Teichert R.W., Bandyopadhyay P., Olivera B.M. (2016). Glycine-rich Conotoxins from the *Virgiconus* clade. Toxicon.

[76] Lebbe E.K., Ghequire M.G., Peigneur S., Mille B.G., Devi P., Ravichandran S., Waelkens E., D’Souza L., De M.R., Tytgat J. (2016). Novel Conopeptides of Largely Unexplored Indo Pacific *Conus* sp. Mar. Drugs.

[77] Echterbille J., Gilles N., Araóz R., Mourier G., Amar M., Servent D., Pauw E.D., Quinton L. (2017). Discovery and characterization of EII B, a new α-conotoxin from *Conus ermineus* venom by nAChRs affinity capture monitored by MALDI-TOF/TOF mass spectrometry. Toxicon.

[78] Hoggard M.F., Rodriguez A.M., Cano H., Clark E., Tae H.S., Adams D.J., Godenschwege T.A., Marí F. (2017). In vivo and in vitro testing of native α-conotoxins from the injected venom of *Conus purpurascens*. Neuropharmacology.

[79] Kauferstein S., Kendel Y., Nicke A., Coronas F.I.V., Possani L.D., Favreau P., Krizaj I., Wunder C., Kauert G., Mebs D. (2009). New conopeptides of the D-superfamily selectively inhibiting neuronal nicotinic acetylcholine receptors. Toxicon.

[80] Jimenez E.C., Olivera B.M. (2010). Divergent M- and O-superfamily peptides from venom of fish-hunting *Conus parius*. Peptides.

[81] Ye M., Hong J., Zhou M., Huang L., Shao X., Yang Y., Sigworth F.J., Chi C., Lin D., Wang C. (2011). A novel conotoxin, qc16a, with a unique cysteine framework and folding. Peptides.

[82] Ye M., Khoo K.K., Xu S., Zhou M., Boonyalai N., Perugini M.A., Shao X., Chi C., Galea C.A., Wang C. (2012). A helical conotoxin from *Conus imperialis* has a novel cysteine framework and defines a new superfamily. J. Biol. Chem..

[83] Xu S., Shao X., Yan M., Chi C., Lu A., Wang C. (2015). Identification of Two Novel O2-Conotoxins from *Conus generalis*. Int. J. Pept. Res. Ther..

[84] Vetter I., Dekan Z., Knapp O., Adams D.J., Alewood P.F., Lewis R.J. (2012). Isolation, characterization and total regioselective synthesis of the novel μO-conotoxin MfVIA from *Conus magnificus* that targets voltage-gated sodium channels. Biochem. Pharmacol..

[85] Inserra M.C., Kompella S.N., Vetter I., Brust A., Daly N.L., Cuny H., Craik D.J., Alewood P.F., Adams D.J., Lewis R.J. (2013). Isolation and characterization of α-conotoxin LsIA with potent activity at nicotinic acetylcholine receptors. Biochem. Pharmacol..

[86] Franco A., Kompella S.N., Akondi K.B., Melaun C., Daly N.L., Luetje C.W., Alewood P.F., Craik D.J., Adams D.J., Mari F. (2012). RegIIA: An alpha 4/7-conotoxin from the venom of *Conus regius* that potently blocks alpha 3 beta 4 nAChRs. Biochem. Pharmacol..

[87] Braga M.C., Nery A.A., Ulrich H., Konno K., Sciani J.M., Pimenta D.C. (2013). α-RgIB: A Novel Antagonist Peptide of Neuronal Acetylcholine Receptor Isolated from *Conus regius* Venom. Int. J. Pept..

[88] Favreau P., Benoit E., Hocking H.G., Carlier L., Hoedt D.D., Leipold E., Markgraf R., Schlumberger S., Córdova M.A., Gaertner H. (2012). A novel µ-conopeptide, CnIIIC, exerts potent and preferential inhibition of NaV1.2/1.4 channels and blocks neuronal nicotinic acetylcholine receptors. Br. J. Pharmacol..

[89] Nguyen B., Caer J.P., Mourier G., Thai R., Lamthanh H., Servent D., Benoit E., Molgó J. (2014). Characterization of a Novel *Conus bandanus* Conopeptide Belonging to the M-Superfamily Containing Bromotryptophan. Mar. Drugs.

[90] Abdel-Wahab M., Miyashita M., Kitanaka A., Juichi H., Sarhan M., Fouda M., Abdel-Rahman M., Saber S., Nakagawa Y. (2016). Characterization of the venom of the vermivorous cone snail *Conus fulgetrum*. Biosci. Biotechnol. Biochem..

[91] Yu S., Du T., Liu Z., Wu Q., Feng G., Dong M., Zhou X., Jiang L., Dai Q. (2016). Im10A, a short conopeptide isolated from *Conus imperialis* and possesses two highly concentrated disulfide bridges and analgesic activity. Peptides.

[92] Johanna B., Samanta J., Javier G.L., Noda F.J., Enrique S., Emilio S., Daniela C., Aguilar M.B., Alexei L.N. (2016). A New Member of Gamma-Conotoxin Family Isolated from *Conus princeps* Displays a Novel Molecular Target. Toxins.

[93] Han T.S., Teichert R.W., Olivera B.M., Bulaj G. (2008). *Conus* venoms—A rich source of peptide-based therapeutics. Curr. Pharm. Des..

[94] Shon K.J., Grilley M.M., Marsh M., Yoshikami D., Hall A.R., Kurz B., Gray W.R., Imperial J.S., Hillyard D.R., Olivera B.M. (1995). Purification, characterization, synthesis, and cloning of the lockjaw peptide from Conus purpurascens venom. Biochemistry.

[95] Santos A.D., Mcintosh J.M., Hillyard D.R., Cruz L.J., Olivera B.M. (2004). The A-superfamily of conotoxins: Structural and functional divergence. J. Biol. Chem..

[96] Mcintosh J.M., Plazas P.V., Watkins M., Gomezcasati M.E., Olivera B.M., Elgoyhen A.B. (2005). A Novel α-Conotoxin, PeIA, Cloned from *Conus pergrandis*, Discriminates between Rat α9α10 and α7 Nicotinic Cholinergic Receptors. J. Biol. Chem..

[97] Yuan D.D., Han Y.H., Wang C.G., Chi C.W. (2007). From the identification of gene organization of conotoxins to the cloning of novel toxins. Toxicon.

[98] Wang Q., Jiang H., Han Y.H., Yuan D.D., Chi C.W. (2008). Two different groups of signal sequence in M-superfamily conotoxins. Toxicon.

[99] Peng C., Liu L., Shao X., Chi C., Wang C. (2008). Identification of a novel class of conotoxins defined as V-conotoxins with a unique cysteine pattern and signal peptide sequence. Peptides.

[100] Yuan D.D., Liu L., Shao X.X., Peng C., Chi C.W., Guo Z.Y. (2008). Isolation and cloning of a conotoxin with a novel cysteine pattern from *Conus caracteristicus*. Peptides.

[101] Bendtsen J.D., Nielsen H., Von H.G., Brunak S. (2004). Improved prediction of signal peptides: SignalP 3.0. J. Mol. Biol..

[102] Luo S., Zhangsun D., Harvey P.J., Kass Q., Wu Y., Zhu X., Hu Y., Li X., Tsetlin V.I., Christensen S. (2015). Cloning, synthesis, and characterization of αO-conotoxin GeXIVA, a potent α9α10 nicotinic acetylcholine receptor antagonist. Proc. Natl. Acad. Sci. USA.

[103] Luo S., Zhangsun D., Wu Y., Zhu X., Hu Y., Mcintyre M., Christensen S., Akcan M., Craik D.J., Mcintosh J.M. (2013). Characterization of a Novel α-Conotoxin from *Conus textile* that Selectively Targets α6/α3β2β3 Nicotinic Acetylcholine Receptors. J. Biol. Chem..

[104] Luo S., Zhangsun D., Zhu X., Wu Y., Hu Y., Christensen S., Harvey P.J., Akcan M., Craik D.J., Mcintosh J.M. (2013). Characterization of a Novel Alpha-Conotoxin TxID from *Conus textile* that Potently Blocks rat Alpha3beta4 Nicotinic Acetylcholine Receptors. J. Med. Chem..

[105] Luo S., Zhangsun D., Schroeder C.I., Zhu X., Hu Y., Wu Y., Weltzin M.M., Eberhard S., Kaas Q., Craik D.J. (2014). A novel α4/7-conotoxin LvIA from *Conus lividus* that selectively blocks α3β2 vs. α6/α3β2β3 nicotinic acetylcholine receptors. FASEB J..

[106] Chen J., Liang L., Ning H., Cai F., Liu Z., Zhang L., Zhou L., Dai Q. (2018). Cloning, Synthesis and Functional Characterization of a Novel α-Conotoxin Lt1.3. Mar. Drugs.

[107] Zhangsun D., Luo S., Wu Y., Zhu X., Hu Y., Xie L. (2010). Novel O-superfamily conotoxins identified by cDNA cloning from three vermivorous *Conus* species. Chem. Biol. Drug Des..

[108] Swa H., Lewis R.J. (2018). Venomics-Accelerated Cone Snail Venom Peptide Discovery. Int. J. Mol. Sci..

[109] Utkin Y.N. (2017). Modern trends in animal venom research—Omics and nanomaterials. World J. Biol. Chem..

[110] Utkin Y.N. (2015). Animal venom studies: Current benefits and future developments. World J. Biol. Chem..

[111] Peng C., Yao G., Gao B.M., Fan C.X., Bian C., Wang J., Cao Y., Wen B., Zhu Y., Ruan Z. (2016). High-throughput identification of novel conotoxins from the Chinese tubular cone snail (*Conus betulinus*) by multi-transcriptome sequencing. Gigascience.

[112] Mardis E.R. (2013). Next-generation sequencing platforms. Annu. Rev. Anal. Chem..

[113] Quail M.A., Miriam S., Paul C., Otto T.D., Harris S.R., Connor T.R., Anna B., Swerdlow H.P., Yong G. (2012). A tale of three next generation sequencing platforms: Comparison of Ion Torrent, Pacific Biosciences and Illumina MiSeq sequencers. BMC Genom..

[114] Safavi-Hemami H., Young N.D., Williamson N.A., Purcell A.W. (2010). Proteomic interrogation of venom delivery in marine cone snails: Novel insights into the role of the venom bulb. J. Proteome Res..

[115] Himaya S.W., Jin A.H., Dutertre S., Giacomotto J., Mohialdeen H., Vetter I., Alewood P.F., Lewis R.J. (2015). Comparative Venomics Reveals the Complex Prey Capture Strategy of the Piscivorous Cone Snail *Conus catus*. J. Proteome Res..

[116] Barghi N., Concepcion G.P., Olivera B.M., Lluisma A.O. (2015). Comparison of the Venom Peptides and Their Expression in Closely Related *Conus* Species: Insights into Adaptive Post-speciation Evolution of *Conus* Exogenomes. Genome Biol. Evol..

[117] Petersen T.N., Brunak S., Von G.H., Nielsen H. (2011). SignalP 4.0: Discriminating signal peptides from transmembrane regions. Nat. Methods.

[118] Lavergne V., Dutertre S., Jin A.H., Lewis R.J., Taft R.J., Alewood P.F. (2013). Systematic interrogation of the *Conus marmoreus* venom duct transcriptome with ConoSorter reveals 158 novel conotoxins and 13 new gene superfamilies. BMC Genom..

[119] Robinson S.D., Safavihemami H., Mcintosh L.D., Purcell A.W., Norton R.S., Papenfuss A.T. (2014). Diversity of Conotoxin Gene Superfamilies in the Venomous Snail, *Conus victoriae*. PLoS ONE.

[120] Kaas Q., Westermann J.C., Halai R., Wang C.K.L., Craik D.J. (2008). ConoServer, a database for conopeptide sequences and structures. Bioinformatics.

[121] Kaas Q., Yu R., Jin A.H., Dutertre S., Craik D.J. (2012). ConoServer: Updated content, knowledge, and discovery tools in the conopeptide database. Nucleic Acids Res..

[122] Magrane M., Martin M.J., O’Donovan C., Apweiler R. (2004). Protein Sequence Databases. Curr. Opin. Chem. Biol..

[123] Consortium U.P. (2015). UniProt: A hub for protein information. Nucleic Acids Res..

[124] Holford M., Zhang M.M., Gowd K.H., Azam L., Green B.R., Watkins M., Ownby J.P., Yoshikami D., Bulaj G., Olivera B.M. (2009). Pruning nature: Biodiversity-derived discovery of novel sodium channel blocking conotoxins from *Conus bullatus*. Toxicon.

[125] Gilly W.F., Richmond T.A., Duda T.F., Elliger C., Lebaric Z., Schulz J., Bingham J.P., Sweedler J.V. (2011). A diverse family of novel peptide toxins from an unusual cone snail, *Conus californicus*. J. Exp. Biol..

[126] Helena S.H., Lu A., Li Q., Fedosov A.E., Jason B., Patrice S.C., Jon S., Mark Y., Olivera B.M. (2016). Venom Insulins of Cone Snails Diversify Rapidly and Track Prey Taxa. Mol. Biol. Evol..

[127] Gao B., Peng C., Lin B., Chen Q., Zhang J., Shi Q. (2017). Screening and Validation of Highly-Efficient Insecticidal Conotoxins from a Transcriptome-Based Dataset of Chinese Tubular Cone Snail. Toxins.

[128] Tayo L.L., Lu B., Cruz L.J., Rd Y.J. (2010). Proteomic analysis provides insights on venom processing in *Conus textile*. J. Proteome Res..

[129] Hu H., Bandyopadhyay P.K., Olivera B.M., Yandell M. (2011). Characterization of the *Conus bullatus* genome and its venom-duct transcriptome. BMC Genom..

[130] Terrat Y., Biass D., Dutertre S., Favreau P., Remm M., Stöcklin R., Piquemal D., Ducancel F. (2012). High-resolution picture of a venom gland transcriptome: Case study with the marine snail *Conus* consors. Toxicon.

[131] Lluisma A.O., Milash B.A., Moore B., Olivera B.M., Bandyopadhyay P.K. (2012). Novel venom peptides from the cone snail *Conus pulicarius* discovered through next-generation sequencing of its venom duct transcriptome. Mar. Genom..

[132] Barghi N., Concepcion G.P., Olivera B.M., Lluisma A.O. (2015). High Conopeptide Diversity in *Conus tribblei* Revealed Through Analysis of Venom Duct Transcriptome Using Two High-Throughput Sequencing Platforms. Mar. Biotechnol..

[133] Jin A.H., Vetter I., Himaya S.W.A., Alewood P.F., Lewis R.J., Dutertre S. (2015). Transcriptome and proteome of *Conus* planorbis identify the nicotinic receptors as primary target for the defensive venom. Proteomics.

[134] Robinson S.D., Li Q., Lu A., Bandyopadhyay P.K., Yandell M., Olivera B.M., Safavihemami H. (2017). The Venom Repertoire of *Conus gloriamaris* (Chemnitz, 1777), the Glory of the Sea. Mar. Drugs.

[135] Domon B., Aebersold R. (2006). Mass spectrometry and protein analysis. Science.

[136] Ueberheide B.M., Fenyö D., Alewood P.F., Chait B.T. (2009). Rapid sensitive analysis of cysteine rich peptide venom components. Proc. Natl. Acad. Sci. USA.

[137] Phuong M.A., Mahardika G.N., Alfaro M.E. (2016). Dietary breadth is positively correlated with venom complexity in cone snails. BMC Genom..

[138] Aebersold R., Mann M. (2016). Mass-spectrometric exploration of proteome structure and function. Nature.

[139] Petras D., Heiss P., Süssmuth R.D., Calvete J.J. (2015). Venom proteomics of Indonesian king cobra, Ophiophagus hannah: Integrating top-down and bottom-up approaches. J. Proteome Res..

[140] Kaas Q., Craik D.J. (2015). Bioinformatics-Aided Venomics. Toxins.

